# XLOS-Observed Mutations of MID1 Bbox1 Domain Cause Domain Unfolding

**DOI:** 10.1371/journal.pone.0107537

**Published:** 2014-09-12

**Authors:** Katharine M. Wright, Kuanlin Wu, Omotolani Babatunde, Haijuan Du, Michael A. Massiah

**Affiliations:** Department of Chemistry, George Washington University, Washington, D.C., United States of America; MRC National Institute for Medical Research, United Kingdom

## Abstract

MID1 catalyzes the ubiquitination of the protein alpha4 and the catalytic subunit of protein phosphatase 2A. Mutations within the MID1 Bbox1 domain are associated with X-linked Opitz G syndrome (XLOS). Our functional assays have shown that mutations of Ala130 to Val or Thr, Cys142 to Ser and Cys145 to Thr completely disrupt the polyubiquitination of alpha4. Using NMR spectroscopy, we characterize the effect of these mutations on the tertiary structure of the Bbox1 domain by itself and in tandem with the Bbox2 domain. The mutation of either Cys142 or Cys145, each of which is involved in coordinating one of the two zinc ions, results in the collapse of signal dispersion in the HSQC spectrum of the Bbox1 domain indicating that the mutant protein structure is unfolded. Each mutation caused the coordination of both zinc ions, which are ∼13 Å apart, to be lost. Although Ala130 is not involved in the coordination of a zinc ion, the Ala130Thr mutant Bbox1 domain yields a poorly dispersed HSQC spectrum similar to those of the Cys142Ser and Cys145Thr mutants. Interestingly, neither cysteine mutation affects the structure of the adjacent Bbox2 domain when the two Bbox domains are engineered in their native tandem Bbox1-Bbox2 protein construct. Dynamic light scattering measurements suggest that the mutant Bbox1 domain has an increased propensity to form aggregates compared to the wild type Bbox1 domain. These studies provide insight into the mechanism by which mutations observed in XLOS affect the structure and function of the MID1 Bbox1 domain.

## Introduction

MID1 is a microtubule-associated protein that belongs to the tripartite motif (TRIM) family. We recently demonstrated that MID1 catalyzes the polyubiquitination of the catalytic subunit of protein phosphatase 2A (PP2Ac) (Du *et al.*, PLoS One, manuscript in review) and the protein alpha4 [1], [2], [3]. PP2Ac is the catalytic component of the heterotrimeric Ser/Thr phosphatase complex that includes the scaffolding (PR65, PP2Aa) and regulatory (PP2Ab) subunits [4], [5], [6]. PP2A regulates cellular processes associated with metabolism, signal transduction, cell-cycle progression and apoptosis [2], [7], [8], [9], [10], [11], [12], [13], [14], [15], [16], [17]. Alpha4 is a novel regulator of PP2A targeting many pathways including the target of rapamycin (TOR) [18], [19], [20]. The ability of MID1 to bind and catalyze the ubiquitination of PP2Ac and alpha4 depends on its zinc-binding RING and Bbox domains.

**Figure 1:**
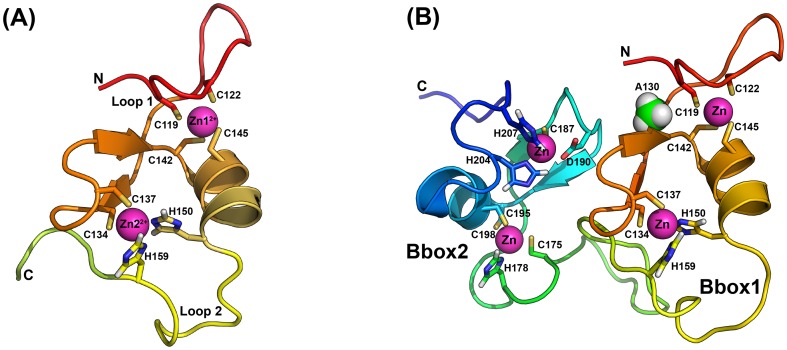
Structure of MID1 Bbox1 and Bbox2 domains. **A.** Ribbon representation of the tertiary structure of the MID1 Bbox1 domain. The Bbox1 domain coordinates two zinc ions and adopts a ββα-RING fold. Amino acid residues coordinating the zinc ions are labeled. Spheres (magenta) represent the two zinc ions. **B**. Ribbon representation of the MID1 Bbox1-Bbox2 domains in tandem. Each Bbox domain adopts a similar ββα-RING fold. The structure of the Bbox1 domain is shown with a similar orientation as the structure of the Bbox1 domain alone in A. The side chain methyl group of Ala130 is shown as spheres.

MID1 has two consecutive zinc-binding Bbox domains that follow the N-terminal RING domain [21]. The two Bbox domains do not share a high degree of sequence similarity or zinc-binding motifs. The first Bbox domain (Type-1, Bbox1) comprises approximately 50-55 residues with the consensus sequence: C-x_(2)_-C-x_(7–10)_C-x_(2)_-C-x_(4–5)_-C-x_(2)_-C(H)-x_(3–6)_-H-x_(2–8)_-H **[C_5_(C/H)H_2_]**. The second Bbox domain (Type-2, Bbox2) is approximately 40-45 amino acids with the consensus sequence: C-x_2-4_-C/H-x_7-10_-C-x_7_-C-x_2_C-x_3-6_-H-x_2-8_-H [**C(C/H)C/DC_3_H_2_**]. The tertiary structures of both types of Bbox domains reveal that each coordinates two zinc ions in a cross-brace manner and adopts the ββα-RING fold typically observed in RING E3 ligases [22], [23], [24], [25], [26] (Figure 1). Subsequently, each Bbox domain is shown to exhibit ubiquitin E3 ligase activity [27], [28].

Thus, MID1 is a novel E3 ligase with three consecutive RING E3 ligase domains. The RING domain exhibits greater E3 ligase activity compared with either Bbox domain [1], [27]. However, the tandem-linked RING-Bbox1-Bbox2 (RB1B2) domains in their natively tethered form show significantly enhanced E3 ligase activity compared with the RING domain alone [27].

Alpha4 binds MID1 via the Bbox1 domain and this interaction is important for the MID1-mediated polyubiquitination of alpha4. Supporting this observation, a Leu146Gln mutation within the Bbox1 domain disrupts the interaction and the polyubiquitination of alpha4 [1]. Similarly, both Bbox domains are required for the polyubiquitination of PP2Ac (Du *et al.*, PLoS One, manuscript in review).

Mutations of MID1 are associated with X-linked Opitz G Syndrome (XLOS), which is characterized by cleft lip/palate, hypertelorism and hyperspadias [2], [29], [30], [31], [32]. Within the Bbox1 domain, residue Ala130 is mutated to a threonine or valine, and the cysteine residues at positions 142 and 145 are mutated to serine and threonine (Cys142Ser, and Cys145Thr), respectively. With each of these mutations, the polyubiquitination of alpha4 is completely disrupted (Du *et al.*, PLoS One, manuscript in review) suggesting that these mutations prevent the interaction of MID1 with alpha4.

Here we used two-dimensional (2D) ^1^H-^15^N NMR spectroscopy to characterize the effects of these mutations on the tertiary structural properties of the MID1 Bbox1 domain. The data reveal that although the mutations involve residues (Ala130, Cys142, Cys145) closely associated in the structure with only one of the two bound zinc ions, the coordination of both zinc atoms is lost, resulting in protein unfolding and aggregation.

## Materials and Methods

### Plasmid construction for recombinant protein production

The DNA encoding the MID1 Bbox1 domain (residues 110–164) and the Bbox1-Bbox2 were cloned into the pET151/D-TOPO vector containing the sequence of a C-terminal His_6_-tag. The Bbox1-Bbox2 construct included residues from the linker region that follow the RING domain (residues 71–110) and is referred to as LB1B2. The Ala130Thr, Ala130Ser, Cys142Ser and Cys145Thr point mutations within the Bbox1 domain were performed using a standard PCR mutagenesis protocol. The sequences of the DNA insert and mutations were confirmed by DNA sequencing before the plasmids were transformed into *E. coli* BL21(*DE3*) cells.

To obtain ^15^N-labeled protein for NMR studies, cells were grown in M9 minimal media supplemented with trace metals, vitamins, glucose and ^15^N-NH_4_Cl. Cells were grown at 37°C to an OD_600_ of ∼0.6 and induced with 0.5 mM isopropyl β-D-1-thiogalactopyranoside for 4 hr at 37°C. Harvested cells were stored at −80°C and then re-suspended in 50 mM Tris (pH 7.7), 200 mM NaCl, 10 mM β-mercaptoethanol, 1 mM ZnCl_2_ and 2% sarkosyl, followed by lysis with sonication [33]. The lysates were clarified by centrifugation at 20,000×*g* at 4°C for 30 min. The proteins were purified using a standard Ni^2+^-NTA affinity chromatography approach. The C-terminal His_6_-tag was not removed because of a lack of a protease cleavage site between the protein and the tag.

### NMR Studies of wild type and mutant Bbox1 constructs

Two-dimensional ^1^H-^15^N heteronuclear single quantum coherence (HSQC) spectra were acquired at 25°C with 0.3 to 0.5 mM ^15^N-labeled wild type and mutant Bbox1 and LB1B2 proteins using a Varian DD2 600 MHz spectrometer equipped with a 5 mm triple resonance (^1^H, ^13^C, and ^15^N) probe with *z*-axis gradient. The NMR data were processed with nmrPipe [34] and visualized with SPARKY [35].

### Dynamic light scattering measurements of wild type and mutant Bbox1

Dynamic light scattering (DLS) data were acquired with a Wyatt Technology DynaPro Nanostar instrument. Three measurements, each with ten acquisitions, were acquired for each protein sample in 100 µL volume. The concentration of the wild type and mutant Bbox1 and Bbox1-Bbox2 proteins was in the range 50–100 nM. DLS data were also acquired for ubiquitin (2 mM) and lysozyme (6.8 mM) to serve as controls. The DLS measurements were benchmarked to predictions of the radius of gyration (R_g_) derived from the corresponding tertiary protein structures using CRYSOL [36] (Table1). Data associated with this manuscript are freely available.

## Results

### Cys142Ser mutation results in a disordered Bbox1 domain

NMR spectroscopy is a highly effective tool in determining the tertiary solution structures of proteins and can be used to evaluate the effect of specific mutations on the structure. A 2D ^1^H-^15^N-HSQC spectrum shows cross peaks that correspond to correlations between directly bonded backbone and side chain ^15^N and H*^N^* atoms. Typically, a spectrum with well-dispersed signals indicates a folded protein while a significant loss in signal dispersion can indicate an unfolded protein lacking a stable tertiary structure [37]. For folded proteins, the H*^N^* and ^15^N signals typically resonate between 7.2–10.2 ppm in the H*^N^* dimension and 102–135 ppm in the ^15^N dimension. In contrast, the corresponding chemical shift dispersion for an unfolded protein can be 7.5–8.3 ppm and 110–125 ppm for the H*^N^* and ^15^N-dimensions, respectively. As a result chemical shift changes in the 2D-^1^H-^15^N-HSQC spectrum can be useful diagnostic for evaluating structural perturbations on the protein structure. Mutations that result in large global structural changes, such as lack of a globular structure, are expected to display drastic changes in peak positions compared to the spectrum of the natively folded protein.

The tertiary structure of the MID1 Bbox1 domain reveals that cysteine residues Cys119, Cys122, Cys142 and Cys145 are coordinated to one of two zinc ions present in the structure (Figure 1). The first pair of cysteine residues is located within loop 1 and Cys142 and Cys145 are found within the first turn of the α-helix. Residues Cys134, Cys137, His150 and His157 coordinate the second zinc ion. Residues Cys134 and Cys137 are also found on loop 1, and the two histidine residues are found on loop 2. The NMR structure reveals that the residues of loop 1 are well ordered [23] while residues of loop 2 show greater mobility [23].

**Figure 2:**
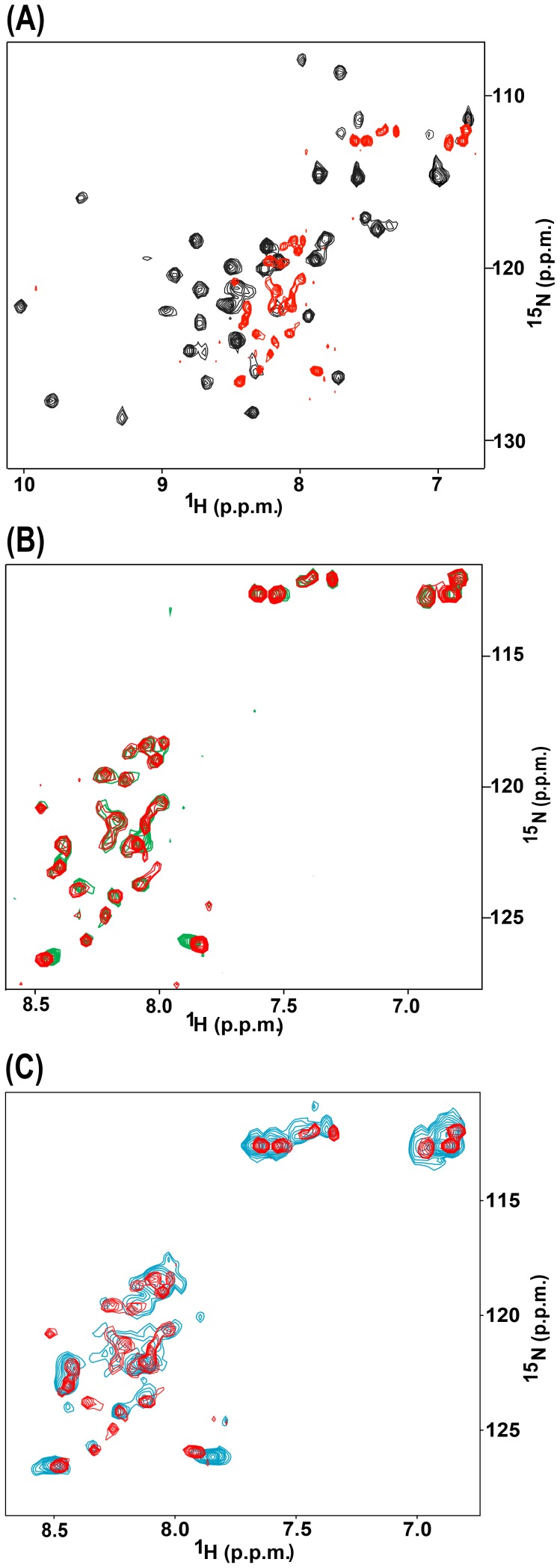
^1^H-^15^N HSQC spectra of the Cys142Ser mutant Bbox1 domain. **A.** Superposition of the HSQC spectrum of wild type MID1 Bbox1 (black) on that of the Cys142Ser mutant Bbox1 domain (red). **B**. Superposition of the HSQC spectrum of Cys142Ser mutant Bbox1 domain in the absence (red) and presence (green) of EDTA. **C**. Superposed HSQC spectra of the wild type Bbox1 domain in the presence of EDTA (blue) and the Cys142Ser Bbox1 domain (red).

To understand how the mutation of Cys142 would affect the structure of the Bbox1 domain, we acquired 2D ^1^H-^15^N-HSQC spectra of the ^15^N-labeled wild type and Cys142Ser mutant Bbox1 domains (MID1 residues 110–164). Figure 2A shows the superposition of these two spectra. The spectrum of the wild type Bbox1 domain shows signals that are sharp and very well dispersed, which is characteristic of a small folded protein. Signals for the H*^N^* and ^15^N atoms were observed over the range of 7.5–10.1 ppm and 105–129 ppm, respectively. In contrast, the cross peaks for the Cys142Ser Bbox1 domain were not well dispersed, with the H*^N^* and ^15^N signals resonating between 7.9–8.5 ppm and 118–127 ppm, respectively (Figure 2A). Furthermore, ∼60% of the H*^N^* signals were not observed at all, most likely due to signal broadening caused by rapid exchange of the H*^N^* atoms with solvent, conformational heterogeneity in the backbone structure, and protein aggregation.

The structure of the wild type Bbox1 domain reveals that Cys142 and the directly coordinated zinc ion are ∼8.5–15 Å from the second zinc ion and its coordinated sphere; the distance between the two zinc ions is 12.8 Å. Therefore, it is possible that the Cys142Ser mutation might only affect the coordination of the first metal ion. To determine whether the coordination of both zinc ions was disrupted, we acquired an HSQC spectrum of 0.4 mM Cys142Ser Bbox1 domain in the presence of 10× molar excess (4 mM) ethylenediaminetetraacetic (EDTA). EDTA is a potent divalent metal chelator. If the Cys142Ser mutant Bbox1 domain retains the ability to coordinate any zinc ions, then EDTA is expected to compete for metal binding and cause additional chemical shift changes in the spectrum. As shown in Figure 2B, the superposition of the spectra of Cys142Ser Bbox1 in the absence and presence of EDTA revealed that the cross peak positions were unaffected. The similarity in chemical shift patterns suggests that the coordination of both zinc ions was already disrupted by the Cys142Ser mutation. Based on the narrow dispersion of the H*^N^* and ^15^N signals, the disappearance of peaks, and the similarity of chemical shifts of peaks in the HSQC spectra in the presence and absence of EDTA, we conclude that the tertiary structure of the Cys142Ser Bbox1 domain is completely disordered.

To confirm that the spectrum of the Cys142Ser Bbox1 domain was consistent with the loss of both zinc ions, and to show that the addition of EDTA to the Cys142Ser mutant (Figure 2B) would have effected the extraction of the second zinc ion if it was still coordinated, we acquired an HSQC spectrum of the wild type Bbox1 domain with the presence of 10× molar excess of EDTA. The spectrum showed collapsed signal dispersion consistent with loss of coordination of both zinc ions and unfolding of the Bbox1 domain. The overlay of the HSQC spectra of the Cys142Ser and wild type Bbox1 domains showed essentially identical chemical shift patterns that almost exactly superpose (Figure 2C).

### Cys145Thr mutation disrupts the structure of the Bbox1 domain but not the Bbox2 domain

We studied the effect of the Cys145Thr mutation on the structure of tandem Bbox1 and Bbox2 domains using the LB1B2 construct (MID1 residues 71–214). Residue Cys145 is coordinated to the same zinc ion as Cys142 and we anticipated that the Cys145Thr mutation would have a similar destabilizing effect on the structure of the Bbox1 domain. Therefore, we used the Cys145Thr mutation to investigate how an unstructured Bbox1 domain would affect the structure of the adjacent Bbox2 domain.

**Figure 3:**
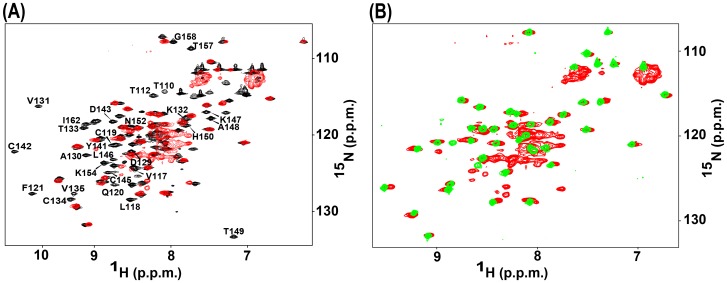
^1^H-^15^N HSQC spectra of the Cys145Thr mutant Bbox1 domain. **A.** Superposed spectrum of wild type LB1B2 (black) on that of the Cys145Thr mutant Bbox1 domain (red). Cross peaks belonging to amino acids of the Bbox1 domain in the wild type folded LB1B2 construct are labeled. Cross peaks for the Cys145Thr Bbox1 domain, which is unfolded, are not readily identifiable. The additional poorly dispersed cross peaks in the center of Figure 3B, compared to the number of poorly dispersed peaks for just the Bbox1 domain shown in Figure 2A, belong to unstructured residues 71–115. **B**. Superposition of the HSQC spectra of Cys145Thr LB1B2 protein (red) and the isolated Bbox2 domain (green). The near exact overlap of signals indicates that the Bbox2 domain within the mutant tandem construct is structured.

The ^1^H-^15^N-HSQC spectrum of the Cys145Thr mutant LB1B2 protein is shown superimposed with that of the corresponding wild type protein in Figure 3A. Many of the ^15^NH signals for the Cys145Thr mutant LB1B2 were collapsed to the center of the spectrum, with poor signal dispersion in both the H*^N^* and ^15^N dimensions. This observation is consistent with the presence of a substantial component of a disordered protein structure, assumed to correspond to the destabilized Bbox1 domain. The additional number of poorly dispersed cross peaks in the center of Figure 3B, compared to the number of poorly dispersed peaks for just the Bbox1 domain shown in Figure 2A, represents the residues that precede the Bbox1 domain in the LB1B2 protein construct (MID1 residues 71–115). There were numerous cross peaks that remained unperturbed and well dispersed, that analysis indicated corresponded to a stably folded Bbox2 domain. To confirm that these signals were from the Bbox2 domain, we compared the spectrum of the Cys145Thr LB1B2 protein to that of the isolated Bbox2 domain (Figure 3B). The majority of dispersed peaks of the Cys145Thr LB1B2 spectrum directly overlapped those of the isolated Bbox2 domain. The relative intensities of this subset of cross peaks were maintained, suggesting that the tertiary structure of the Bbox2 domain was largely unperturbed within the mutated LB1B2 construct.

### Ala130Thr mutation disrupts the tertiary structure of the Bbox1 domain

Residue Ala130 is highly conserved in Bbox1 domain sequences, particularly in the C-I subclass of TRIM proteins consisting of COS, FNIII and B30.2 C-terminal domains [1], [38]. Unlike Cys142 and Cys145, residue Ala130 is not involved in metal coordination. Therefore it was not immediately clear how substitution at this position might affect the tertiary structure of the Bbox1 domain. We investigated the structural effect of the Ala130Thr mutation within the Bbox1 domain both as an isolated protein and in the tandem LB1B2 construct.

**Figure 4:**
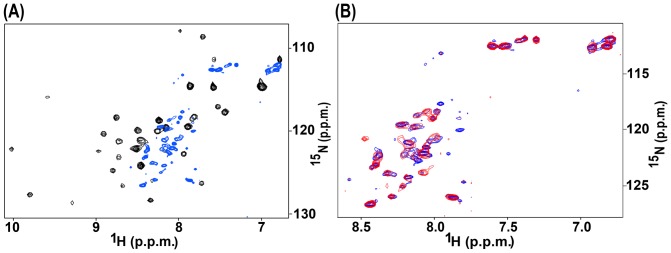
^1^H-^15^N HSQC spectra of the Ala130Thr mutant Bbox1 domain. **A.** Superposed HSQC spectra of wild type Bbox1 domain (black) and the Ala130Thr mutant Bbox1 domain (blue). **B**. Superposition of the HSQC spectra of Ala130Thr (blue) and Cys142Ser (red) mutant Bbox1 domains.

The HSQC spectrum of the Ala130Thr Bbox1 domain showed ^15^N–H cross peaks with very little chemical shift dispersion in either dimension (Figure 4A). Comparison with the spectrum of the wild type Bbox1 domain revealed that the changes were consistent with loss of coordination of both zinc ions. To verify that the Ala130Thr mutation might have a similar structural consequence as the Cys142Ser mutation, we compared the HSQC spectra of the Ala130Thr and Cys142Ser mutant Bbox1 domains (Figure 4B). With the exception of only a few cross peaks, there was good overlap between the two spectra indicating that both mutations caused a similar structural change. Although Ala130 is not involved in zinc coordination it is clear from the spectra that its mutation disrupts the coordination of both zinc ions.

### Bbox1 Ala130Ser mutation perturbs the structure but maintains some functionality

**Figure 5:**
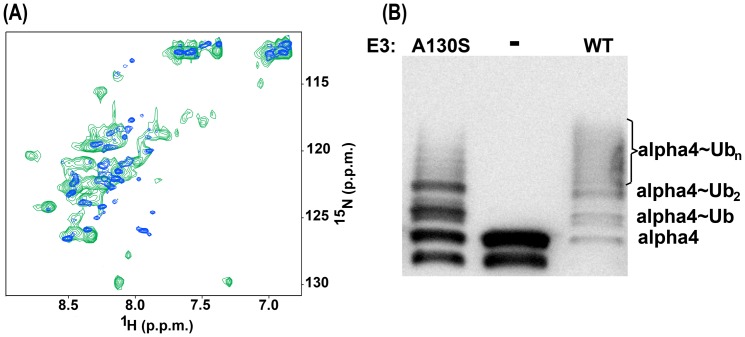
Structural and functional effects of the Ala130Ser mutant Bbox1 domain. **A.** Superposition of the HSQC spectra of Ala130Ser (green) and the Ala130Thr Bbox1 domains (blue). **B**. Western blot showing ubiquitination of alpha4 by the wild type RING-Bbox1-Bbox2 (RB1B2) domain construct and RB1B2 harboring the Ala130Ser mutation. Lane 2 shows the result of a control reaction in which the E3 ligase was omitted. Two strong bands indicating mono- and di-ubiquitinated alpha4 as well as a smearing pattern indicative of polyubiquitinated alpha4 was observed for the Ala130Ser Bbox1 domain.

To understand the role of the side chain groups of threonine at residue position 130, we mutated the Ala130 to a serine; the serine side chain is smaller than threonine, and lacks the methyl group. The HSQC spectrum of the Ala130Ser mutant Bbox1 domain showed poorer dispersion of signals, particularly in the H*^N^* dimension (7.4–8.7 ppm) compared to that of the wild type Bbox1 domain. However compared to the spectrum of the Ala130Thr Bbox1 domain, more signals were observed and the peaks were slightly more spread out (Figure 5A). We acquired an HSQC spectrum of the Ala130Ser Bbox1 domain in the presence of EDTA to determine whether the protein was coordinated to zinc ions. We observed additional chemical shift changes consistent with elimination of metal coordination, similar to the results obtained for EDTA treatment of the wild type Bbox1 domain (Figure 2C). This observation suggests that the Ala130Ser mutant maintains some ability to coordinate zinc.

Because the H*^N^* chemical shift dispersion for the Ala130Ser mutant was reduced, suggestive of a structural perturbation, we decided to test the ability of the RING-Bbox1-Bbox2 (RB1B2) construct harboring the Ala130Ser substitution to catalyze the ubiquitination of alpha4. As shown in Figure 5B, the RB1B2-Ala130Ser construct was able to catalyze the polyubiquitination of alpha4. In contrast, the RB1B2 proteins with the Cys142Ser, Cys145Thr and Ala130Thr/Val mutations were unable to catalyze the polyubiquitination of alpha4 (Du *et al.*, PLoS One, manuscript in review).

### Ala130Thr mutation disrupts the tertiary structure of the Bbox1 domain but not the Bbox2 domain

We investigated the structural effects of the Ala130Thr mutation on the adjacent Bbox2 domain within the LB1B2 protein construct. According to the tertiary structure of the Bbox1-Bbox2 domains in tandem, residue Ala130 is located relatively close to the interface of the two domains (Figure 1B) [39]. Thus, it is possible that the Ala130Thr mutation might affect the structure of the Bbox2 domain.

**Figure 6:**
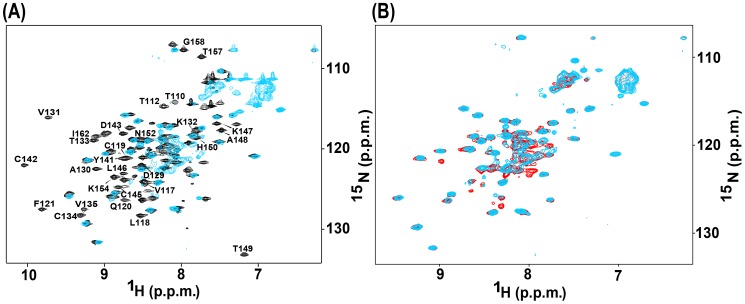
^1^H-^15^N-HSQC spectra of the Ala130Thr mutant Bbox1 domain in tandem LB1B2. **A.** Superposition of the spectrum of Ala130Thr LB1B2 (cyan) on that of wild type LB1B2 (black). Cross peaks for amino acids in the wild type Bbox1 domain are labeled on the wild type LB1B2 spectrum. **B**. Superposition of the spectra of the Ala130Thr domain (cyan) and Cys145Thr (red) LB1B2 protein (red).

We compared the HSQC spectra of the wild type and Ala130Thr LB1B2 proteins (Figure 6A). The spectrum of the Ala130Thr LB1B2 mutant showed a similar pattern of cross peaks to that of the Cys145Thr LB1B2 mutant (Figure 6B). The peaks that lost their chemical shift dispersion corresponded to peaks belonging to residues of the Bbox1 domain. The dispersed signals that remained belong to residues from the Bbox2 domain. This observation suggests that the Ala130Thr and Cys145Thr mutations have a similar structural effect.

### Mutations promote Bbox1 domain aggregation

Natural mutations of MID1 are shown to form cytoplasmic clumps in cells, instead of its normal association with microtubules [2], [21], [40], [41], [42]. Most of these mutations are located within the C-terminal region of MID1. Little is known about how mutations within the N-terminal Bbox1 domain might affect the aggregation state of MID1.

We acquired DLS data of the individual wild type and mutant Bbox1 domain proteins (MW 6 kDa) alone and in tandem with the Bbox2 domain (LB1B2, MW 14 kDa). As a benchmark, we acquired DLS data for ubiquitin (8 kDa) and lysozyme (14 kDa). To validate the radius of gyration (R_g_) derived from the DLS measurements, we utilized the program CRYSOL [36] to predict the R_g_ based on the known tertiary structures of these proteins (Table 1). As expected, the DLS measurements indicated that ubiquitin and lysozyme exist as monomers in solution; in each case the R_g_ was similar to that predicted from the tertiary structure. The DLS measurements of the wild type Bbox1 domain indicated that it might exist as a dimer because the derived R_g_ was twice the predicted R_g_ from the structure. The data for the LB1B2 protein indicated it might be a monomer. In contrast, the DLS output indicated that ∼75% of the population of the Ala130Thr Bbox1 protein in solution had a R_g_ of 35±13 Å, which is roughly three times the predicted value (12 Å). Furthermore, the data indicated that 25% of the population of the Ala130Thr Bbox1 protein formed very large soluble aggregates (R_g_ >300 Å). Similarly, the DLS data suggested that the Cys142Ser Bbox1, Ala130Thr LB1B2, and Cys145Thr LB1B2 proteins could be in equilibrium between dimeric and larger soluble aggregate states. Approximately 70% of the population of the Ala130Ser Bbox1 mutant protein had a R_g_ of ∼18±7 Å, consistent with a monomer-dimer equilibrium.

**Table 1:** Radius of gyration (R_g_) of Bbox1 and Bbox1-Bbox2 domains.

	R_g_(Å) by DLS	% Observed^a^ by DLS	R_g_^b^ (Å)
Ubiquitin (1UBQ)	10.4±1	99.4±0.2	12
Lysozyme (2CDS)	8±1	99.5±0.3	15
Bbox1 WT (2FFW)	25±3	98±2	12
A130T Bbox1	35±13	75±32	—^c^
C142S Bbox1	30±3	62±10	— ^c^
A130S Bbox1	18±7	71±22	— ^c^
LB1B2 WT (2JUN)	22±8	97±3	15
A130T LB1B2	32±3	90±4	— ^c^
C145T LB1B2	33±17	70±28	— ^c^

a. Percentage of the population of protein in solution that accounts for measured R_g_ value.

b. Radius of gyration was predicted using the tertiary structures of these proteins with CRYSOL.

c. The R_g_ could not be predicted for these samples because the structure in the (partially) unfolded state is not known.

## Discussion

The MID1 Bbox1 domain is essential for the polyubiquitination of alpha4 and PP2Ac (Du *et. al*., PLoS One, Manuscript in review [1]). Mutations of the Bbox1 domain are associated with the following midline birth anomalies: hypertelorism, hypospadias, clefts of the lip and/or palate (CL/P), and laryngotracheoesophageal (LTE) cleft. Here we provide structural data indicating that XLOS-observed point mutations within the Bbox1 domain (Ala130Thr, Cys142Ser, Cys145Thr) disrupt the coordination of two structurally critical zinc ions and lead to a failure of the structure to fold correctly.

The mutation of Cys142 to serine replaces the Cys thiol moiety with a hydroxyl group, a relatively conservative mutation. Similarly, the mutation of Cys145 to threonine yields a side chain with a hydroxyl group. Historically, serine and threonine residues are not observed to directly coordinate zinc ions in zinc-finger proteins [43], [44]. A possible reason may be associated with the pK_a_ of these amino acids: the pK_a_ of Cys thiols are typically in the range ∼8–9 while those of Ser hydroxyl groups are 13–15, indicating that it is more difficult to deprotonate the hydroxyl group than the thiol proton. Another reason is that metal-ligand coordination is influenced by the relative polarizability of the ligands. Zinc ions have preference for the nitrogen and sulfur atoms of histidine and cysteine residues, respectively.

The coordination of the two zinc ions, with K*_d_* in the range of 10^−9^–10^−12^ M [45], [46], is essential for the stabilization of the tertiary structure of the Bbox1 domain. Removal of the bound zinc ions, either with EDTA chelation or through mutation of the zinc-binding residues, would be expected to result in the loss of tertiary structure [45], [47]. In our studies, the mutation of Cys142Ser and Cys145Thr led to failure of the Bbox1 domain to fold into a globular structure. While it is to be expected that mutation of these zinc-binding cysteine residues would disrupt the coordination of the directly attached zinc ion, it is unclear that the loss of coordination of that zinc ion would cause the binding of the other zinc ion, which is ∼13 Å away, to also be disrupted. According to the tertiary structure of the Bbox1 domain, the loss in coordination of the zinc bound by Cys residues119, 122, 142 and 145 would lead to increased mobility of loop 1, which in turn would destabilize the positions of the two other cysteine residues and prevent binding the second zinc ion.

**Figure 7:**
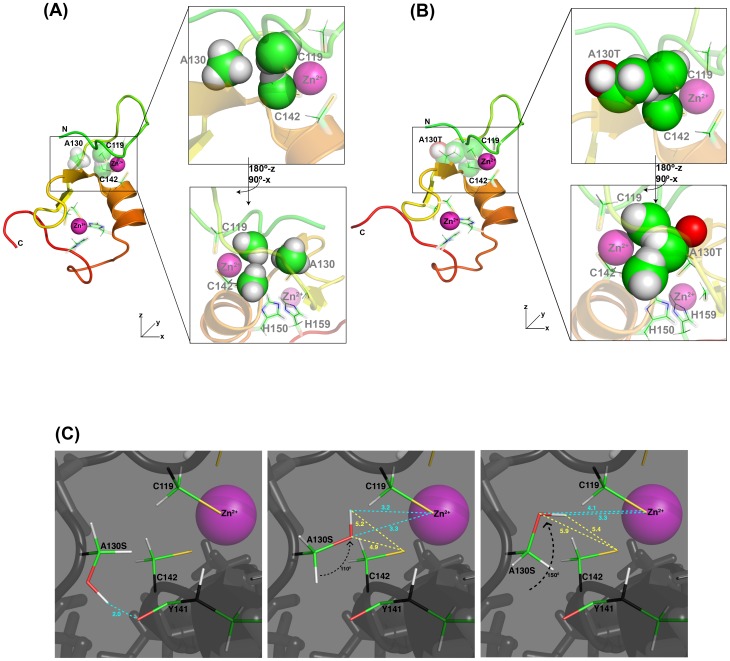
Model of the A130 mutants. **A.** Ribbon drawing of MID1 Bbox1 domain with sphere representation for the side chain atoms of Ala130, Cys119 and Cys142. Top right: a close-up view of these three residues. Bottom right: a top-down view with the molecule rotated as indicated to highlight the position of the methyl group of Ala130 relative to the methylene atoms of the two cysteine residues. **B**. Views from the same orientation as in A showing a model in which a threonine replaces Ala130. The space-filling model reveals that the methyl group of the threonine would clash with the methylene atoms of Cys119 and Cys142. **C**. Close-up views of the position of the side chain locations in a model of the Ala130Ser mutation. Shown is a large cavity and that could accommodate each of three possible rotameric states of the serine side chain. As a result, the serine hydroxyl group could be involved in different hydrogen-bonds. The figures were generated with Pymol.

The loss of signal dispersion in the HSQC spectrum of each of the two Cys mutants allows us to understand the structural effect of the Ala130Thr/Ser mutations. The methyl group of Ala130 is ∼3–5 Å from the backbone and methylene group of both Cys119 and Cys142 (Figure 7A). To understand how the Ala130Thr mutation might affect the Bbox1 domain structure, we modeled a threonine in place of Ala130 and observed the methyl group to be 1.4 Å closer to the methylene atoms of both Cys119 and Cys142, hindering the proper coordination of the zinc ion by the thiol groups (Figure 7B).

The mutation of Ala130 to serine, which was meant to test whether steric clash involving the bulkier threonine side chain is a factor in the disruption of the zinc coordination, results in a spectrum that indicates structural change in the Bbox1 domain (Figure 5A). The use of EDTA reveals that the Ala130Ser mutant Bbox1 domain still coordinates zinc ions. These results and the observation that the Ala130Ser mutant catalyzing the polyubiquitination of the protein alpha4, which requires a functional Bbox1 domain, suggests that the Ala130Ser mutant Bbox1 domain is still folded and can bind alpha4 (Figure 5B). Examination of the structure of the Bbox1 domain reveals that there is a pocket between the methyl group of Ala130 and Cys119 and Cys142. This pocket is large enough to accommodate the serine side chain and its various rotameric states (Figure 7C). Depending on the rotamer of the side chain, we speculate that the hydroxyl group could form a strong hydrogen bond with the backbone carbonyl of Tyr141 or a normal hydrogen bond with the thiol group of Cys119 that could stabilize the modified structure and maintain zinc coordination.

When expressed in the context of a Bbox1-Bbox2 tandem construct (MID1 (residues 71–164), all three mutations affected only the structure of the Bbox1 domain. Even though Ala130 is located near the interface, its mutation contributes little to the Bbox2 domain stability since the structure of the Bbox2 domain remains unperturbed. As such, it is possible that full-length MID1 harboring Bbox1 mutations could still maintain some functionality that is independent of the Bbox1 domain. For example, the RING-Bbox1-Bbox2 (RB1B2) constructs harboring the various Bbox1 domain mutations were capable of catalyzing the polyubiquitination of PP2Ac (Du *et al*, PLoS One, manuscript in review) indicating that the Bbox2 domain can compensate for the loss of function of the Bbox1 domain.

In summary, we used NMR spectroscopy to demonstrate that a number of XLOS-observed mutations within the Bbox1 domain cause the structure of the Bbox1 domain to unfold. The mutations also cause the isolated Bbox1 and Bbox1-Bbox2 proteins to form soluble aggregates. It is therefore conceivable that within the full-length MID1 these mutations might contribute to the formation of cytoplasmic clumps observed in XLOS-derived fibroblasts [40], [48].
